# Relationship between Divergent Thinking and Intelligence: An Empirical Study of the Threshold Hypothesis with Chinese Children

**DOI:** 10.3389/fpsyg.2017.00254

**Published:** 2017-02-22

**Authors:** Baoguo Shi, Lijing Wang, Jiahui Yang, Mengpin Zhang, Li Xu

**Affiliations:** ^1^Beijing Key Laboratory of Learning and Cognition and Department of Psychology, Capital Normal UniversityBeijing, China; ^2^Beijing G&G Human Resource Development CenterBeijing, China

**Keywords:** divergent thinking, intelligence, the threshold hypothesis, creativity, openness to experience

## Abstract

The threshold hypothesis is a classical and notable explanation for the relationship between creativity and intelligence. However, few empirical examinations of this theory exist, and the results are inconsistent. To test this hypothesis, this study investigated the relationship between divergent thinking (DT) and intelligence with a sample of 568 Chinese children aged between 11 and 13 years old using testing and questionnaire methods. The study focused on the breakpoint of intelligence and the moderation effect of openness on the relationship between intelligence and DT. The findings were as follows: (1) a breakpoint at the intelligence quotient (IQ) of 109.20 when investigating the relationship between either DT fluency or DT flexibility and intelligence. Another breakpoint was detected at the IQ of 116.80 concerning the correlation between originality and intelligence. The breakpoint of the relation between the composite score of creativity and intelligence occurred at the IQ of 110.10. (2) Openness to experience had a moderating effect on the correlation between the indicators of creativity and intelligence under the breakpoint. Above this point, however, the effect was not significant. The results suggested a relationship between DT and intelligence among Chinese children, which conforms to the threshold hypothesis. Besides, it remains necessary to explore the personality factors accounting for individual differences in the relationship between DT and intelligence.

## Introduction

Creativity is recognized as the ability to generate ideas or products with novelty and usefulness (Mumford, 2003; Plucker et al., 2004). This ability has often been considered a key competency of a member of society with significant value to social progress (Jauk et al., 2015). According to Guilford (1967), the core of creativity is divergent thinking (DT), which is the ability of an individual to generate as many answers as possible to a problem. As a result, DT tasks have long been employed to evaluate people’s creativity (Sayed and Mohamed, 2013), and DT tests have become the most popular psychometric assessment tools in creativity research fields (Acar and Runco, 2014). Previous studies indicate that tests of DT are reliable and valid predictors of certain creative performance criteria, although they do not guarantee actual creative achievement (Runco and Acar, 2012). They are effective measurements to assess creative ability (Colzato et al., 2012). As one of the most well-constructed tests to assess creativity (Zhu et al., 2013), the Torrance Test of Creative Thinking (TTCT) is often employed in studies, including this study.

Intelligence as a strong predictor of educational and occupational achievements has been investigated for more than 100 years. According to the construct introduced by Cattell (1963), general crystallized intelligence (Gc) and general fluid intelligence (Gf) are two central constructs in the concept of intelligence. Gc represents the breadth and depth of a person’s knowledge and abilities to use this knowledge. Conversely, Gf represents the ability to employ a type of mental operation to independently reason and solve novel problems; it does not benefit from acquired experience (Sawyer, 2012). Gf is critical for an extensive variety of cognitive activities and is considered to be one of the most important aspects in the learning process. It is closely related to success of career and life, especially in the contemporary complex social environments (Jaeggi et al., 2008). Among many of the evaluation tools, the Raven’s Progressive Matrices (RPM) is one of the most extensively employed tests (Zhang and Wang, 1989). This test seeks to directly measure the general cognitive ability or educative ability, which is the ability to make meaning out of confusion and to make rational judgments (Raven, 2000), and provide an evaluation of non-verbal ability. Many studies use Raven’s Standard Progressive Matrices (RSPM) to measure fluid intelligence (Bilker et al., 2012; Downey et al., 2014); this measurement shows high reliability and validity. In this study, we also employ RSPM to assess fluid intelligence.

As two important components of giftedness, creativity and intelligence are both individual differences that explain why a person has higher potential than others to generate solutions to problems (Sternberg, 2008). Creativity enables people to think about things in a novel manner and facilitate the development of civilization, whereas intelligence helps people solve problems in a logical manner. Over the last six decades, intelligence has received substantially more academic attention than creativity (Batey et al., 2010). However, the relationship between creativity and intelligence remains unclear (Kaufman and Plucker, 2011).

According to Sternberg and O’Hara (1999), five types of possible relationships between creativity and intelligence exist: the former is a subset of the latter; the latter is a subset of the former; both variables are overlapping sets; both variables are fundamentally the same (coincident sets); and both variables are unrelated (disjoint sets). Considering that the last two types of relationships (coincident and disjoint sets) are very rare (Plucker et al., 2015), the remaining three relationships should be carefully scrutinized. Guilford’s (1967) work on the structure of the intellect (SOI) implies that creativity is a subset of intelligence; he considered DT as one of the cognitive operations in the SOI model. Sternberg’s (1997) theory of successful intelligence included creative intelligence as one of the three intelligence components (the remaining two components were analytical intelligence and practical intelligence). Conversely, Amabile’s (1996) componential theory of creativity insists that creativity requires three components: domain-relevant skills (which include intelligence), creativity-relevant skills, and task motivation. Similarly, Sternberg and Lubart’s (1992) investment theory of creativity considers intelligence to be one of six necessary elements that help to produce creativity. The third relationship between creativity and intelligence is that the two constructs have some overlapping sets and differences. According to the three-ring theory of giftedness proposed by Renzulli (1978), a gifted individual has both high intellectual ability and high creativity; intelligence and creativity significantly overlap in this meaning. All previously mentioned theories indicate a close relationship between intelligence and creativity. Therefore, the focus is how the two variables are related (Plucker and Renzulli, 1999).

Of all explanations, the threshold hypothesis (Getzels and Jackson, 1962; Guilford, 1967; Fuchs-Beauchamp et al., 1993) is a classical and notable hypothesis. According to this theory, the relationship between creativity and intelligence may vary at different levels of intelligence. Guilford and Christensen (1973) assumed a break in the correlation data between intelligence quotient (IQ) and creativity at an IQ level of approximately 120. Below an IQ level of 120, a correlation between IQ and creativity is observed, whereas no correlation is observed at IQ levels above 120. The basic idea of the threshold hypothesis means that high creativity requires high intelligence or above-average intelligence. Above-average intelligence is considered to form a necessary but insufficient condition for high creativity (Guilford, 1967). People with intelligence below average intelligence have little chance of being very creative; those with intelligence above the threshold may have the potential of high creativity but it is not related to their IQ level.

As Plucker et al. (2015) suggested, many theoretical treatments of the creativity-intelligence link exist compared with few empirical studies. Among them, only a few in fact have systematically examined the threshold hypothesis and conclusions are inconsistent (Runco, 1991). Through the study of a gifted sample, Barron (1963, 1969) observed intelligence had no significant correlation with creativity but a significant correlation did exist in a sample of average intelligence. Schubert (1973) and Weinstein and Bobko (1980) discovered that the relationship between intelligence and creativity varies with different levels of IQ and intelligence has a weaker correlation with creativity in the top half of the intelligence range than in the bottom half of the intelligence range. In addition, with a sample of 338 gifted (IQ ≥ 130) and 220 non-gifted (IQ < 130) 7.8–14-year-old children, Guignard et al. (2016) indicated that the threshold effect was only found for correlations between verbal integrative thinking and perceptual reasoning or processing speed. In contrast to these points, some studies provide evidence that does not support the threshold hypothesis. For example, Runco and Albert (1986) used California Achievement Test (CAT) scores as the estimate of intelligence and discovered that the coefficient between DT and the CAT was very significant in the high ability group with fifth to eighth grader students; this result refuted the threshold hypothesis. Preckel et al. (2006) examined the relationship between DT and fluid intelligence with a sample of 1328 German 12–16 years old students and discovered that correlations between both variables are almost equal at different IQ levels.

Moreover, a problem of a common measure applied to investigate the threshold hypothesis was mentioned in related research recently. In previous studies, researchers tested the threshold hypothesis by dividing a sample at a given level (e.g., at 120 IQ points) and separately estimated the correlations for lower and higher IQ groups. However, empirical studies cannot prove that the threshold should be defined as 120 IQ points (Jauk et al., 2013). Based on this consideration, several very recent studies examined the threshold using different data analysis techniques. For example, Karwowski and Gralewski (2013) observed a threshold of 115 IQ points in a sample of Polish middle school students after applying item response theory and confirmatory factor analysis. Jauk et al. (2013) examined the threshold of intelligence in a sample of adult participants by a segmented regression analysis and obtained a threshold of 104.00 (or 119.60) IQ points for the originality of two (or many) original ideas and a threshold of 86.09 IQ points for ideational fluency. For a particularly advanced indicator such as creative achievement, no threshold effect was observed, which suggests that intelligence is linked with creative achievement across the entire IQ range. Mourgues et al. (2016) explored the threshold theory with 4368 third to eleventh grade Saudi Arabian students, and only for sixth to eighth (108.8) and ninth to eleventh (108.4) graders the thresholds were detected.

Instead of setting a threshold prior to statistical analysis, techniques such as segmented regression can detect the breakpoint in continuous data (Jauk et al., 2013), which enables us to obtain a natural threshold of intelligence (rather than one artificially set in advance). Furthermore, Karwowski et al. (2016) adopted a recently developed methodology, the Necessary Condition Analysis (NCA) (Dul, 2016), corroborating the necessary-but-not-sufficient relationship between intelligence and creativity in eight studies. Based on these analyses, the current study performed these two empirical methods to explore the relationship between creativity and intelligence. Considering that previous studies explored the threshold effect of participants in Western culture (e.g., Runco and Albert, 1986; Preckel et al., 2006; Jauk et al., 2013; Welter et al., 2016), this effect should be examined using Chinese subjects in Eastern culture. As many previous studies indicated, compared with their counterparts in Western developed countries, Chinese adolescents and children demonstrated significantly lower performance in creativity (e.g., Ye et al., 1988; Hu et al., 2004). However, numerous other studies revealed that Chinese subjects were not inferior in intelligence to Western subjects (Lynn et al., 1988, 1991; Jensen and Whang, 1993). To explain this inconsistent phenomenon, intelligence likely contributes less to creativity among Chinese children, and the threshold of intelligence (if it does exist) will be lower than the threshold of intelligence among Western children (which is typically hypothesized as 120 points). The variation in the threshold effect of intelligence for Chinese children should be examined considering the cultural differences between Chinese children and Western children.

Studies of the association between personality traits and DT have been conducted using different samples in recent years (Gelade, 2002). Of these personality factors, openness to experience is viewed as the most important factor for creativity (Furnham, 1999; Chamorro-Premuzic and Furnham, 2004) because it is consistently linked with all criteria of creativity (Kerr and McKay, 2013). Some recent studies investigated the role of openness to experience in the intelligence–creativity relationship. In a questionnaire study with high school students, Ivcevic and Brackett (2015) identified a significant interaction between emotional regulation ability and openness to experience. This result revealed that openness can moderate the relationship between emotional intelligence (measured by emotional regulation ability) and creativity. Shi et al. (2016) examined the moderating effect of openness to experience between intelligence and DT with 831 children; the results indicated a significant moderate effect. Intelligence was closely associated with creativity when an individual had a medium openness or high openness to experience. Openness to experience served a “catalyst” role (McCrae and Ingraham, 1987) between intelligence and creativity. An examination of whether this enhancing effect of openness consistently exists at different levels of intelligence is necessary.

This research aimed to verify the possible threshold in the intelligence–creativity link with Chinese children via testing and questionnaire methods and an analysis that employed a subsection linear regression model and the NCA. We also examined the moderating role of openness to experience and focused on how it affected the correlation between intelligence and creativity.

## Materials and Methods

### Participants

This study involved 568 primary school students as participants (female = 265). The mean age for fifth graders is 11.2 years old, and the mean age for sixth graders is 12.4 years old. Informed consent was provided by the parents and teachers of these children prior to their participation in accordance with the Declaration of Helsinki. The children’s participation was voluntary. The Research Ethics Board at Capital Normal University approved this study.

### Materials

#### The Measure of DT

A revised version of the TTCT (Torrance, 1966, 1993) was employed in this study to measure DT. This test contains four tasks. For the first task, participants view an elf in a picture and pose as many questions as possible about the scene. For the second task, a picture with an unclear subject is presented to the students, who need to interpret it using as many different nouns as they can. For the third task, participants are required to add lines to 30 pairs of parallel lines to finish many novel and meaningful drawings. For the last task, they are asked to complete ten unfinished drawings by adding lines. Tasks 1, 3, and 4 are selected from the TTCT and have high reliability and validity. Task 2 was developed by the researchers and employed in previous studies (Shi et al., 2012, 2016). The participants complete this creativity test in 30 min.

According to the scoring criteria of the TTCT and Hu et al. (2004), each appropriate answer was individually assessed for fluency (number of responses), flexibility (number of response categories) and originality (percentage of responses, two points for the percentage below 5%, one point for the percentage of 5–10%, and zero points for the percentage higher than 10%). A composite score of DT was acquired by transforming all three criteria to standardized scores and then summing them. Two individual raters scored these items for 50 subjects, and the inter-rater reliabilities ranged from 0.88 to 0.92.

#### The Measure of Intelligence

Intelligence was assessed by a 60-item Chinese version of Raven’s Standard Progressive Matrices (RSPM-CR), with the highest total row score being 60. The reported IQ reflected a standardized score that was converted using the formula of IQ_i_ = 100+15(X_i_-MEAN)/SD, where MEAN is the mean of the normative sample from the Chinese norm (Zhang and Wang, 1989), SD is the standard deviation of the same sample, and X_i_ is the raw score of respondent i. This test was revised based on 5,108 Chinese subjects with ages ranging from 5.5 to over 70 by Zhang and Wang (1989). Its split-half reliability was 0.95 and the coefficient with the Chinese version of Wechsler Intelligence Scale for Children- Revised (Lin and Zhang, 1986) was 0.71.

#### The Measure of Openness to Experience

The Small Five Personality Scale developed by Zhou et al. (2000) is employed to measure the personality factors of the participants. Specifically, we only used the subscale of openness to experience, which includes 26 items, such as “like to indulge in imagination or daydreaming.” It has good reliability (with a Cronbach’s α coefficient of 0.878) and concurrent validity (*r* = 0.659 with the NEO-PI-R, Costa and McCrae, 1992). Participants read each item and rate the extent to which these statements are consistent with their actual situation using a 5-point scale, on which 1 represents *not at all true* and 5 represents *always true*. The total score of this instrument ranges from 26 to 130.

### Procedure

With the guidance of test administrators in each classroom, participants were asked to finish two paper–pencil tests (DT test and RSPM-CR test), respectively, in the first two classes, followed by the openness scale. Approximately 80 min were required to finish all tasks.

### Data Management and Analysis

Little’s missing completely at random (MCAR) test indicated that the data were MCAR (*X*^2^ = 203.67, *df* = 306, *p* = 1.00). Thus, we adopted the mean substitution (MS) method to impute the missing data (Little and Rubin, 2002). Regarding the outliers, the values in the range that were 3 standard deviations from the mean of each variable were excluded.

To detect the breakpoint of intelligence, the segmented regression analyses were implemented via segmented packages in R 3.3.0 (Mueggo, 2008). IQ acts as an independent variable, and the factors of DT (fluency, flexibility, originality, and the composite score) act as dependent variables. The algorithm was supplied with an initial guess parameter (IQ = 100) for the breakpoints according to Jauk et al. (2013). Then, NCA (Dul, 2016; Karwowski et al., 2016) was conducted to reanalyze the data, through which we could obtain the necessity effect size.

## Results

### Descriptive Statistical Analysis

The mean and standard deviation of each variable and the correlation coefficients are listed in **Table 1**. Significant positive correlations existed among the variables, which is consistent with expectations; thus, we can expand additional statistical tests to explore the relationships among the variables.

**Table 1:** Means (M), standard deviations (SD), and correlations of the variables.

	*M*	*SD*	1	2	3	4	5	6
1 Intelligence	104.69	10.54	-					
2 Openness	92.43	14.36	0.23^∗∗∗^	-				
3 Fluency	8.16	3.29	0.37^∗∗∗^	0.39^∗∗∗^	-			
4 Flexibility	5.66	1.84	0.40^∗∗∗^	0.38^∗∗∗^	0.93^∗∗∗^	-		
5 Originality	4.72	4.82	0.34^∗∗∗^	0.42^∗∗∗^	0.83^∗∗∗^	0.77^∗∗∗^	-	
6 Composite score of creativity	0.00	2.84	0.39^∗∗∗^	0.42^∗∗∗^	0.97^∗∗∗^	0.95^∗∗∗^	0.92^∗∗∗^	-

*N = 568; ^∗∗∗^*p* < 0.01, two-sided test.*

### Segmented Regression Analyses of the Relationship between DT and Intelligence

**FIGURE 1:**
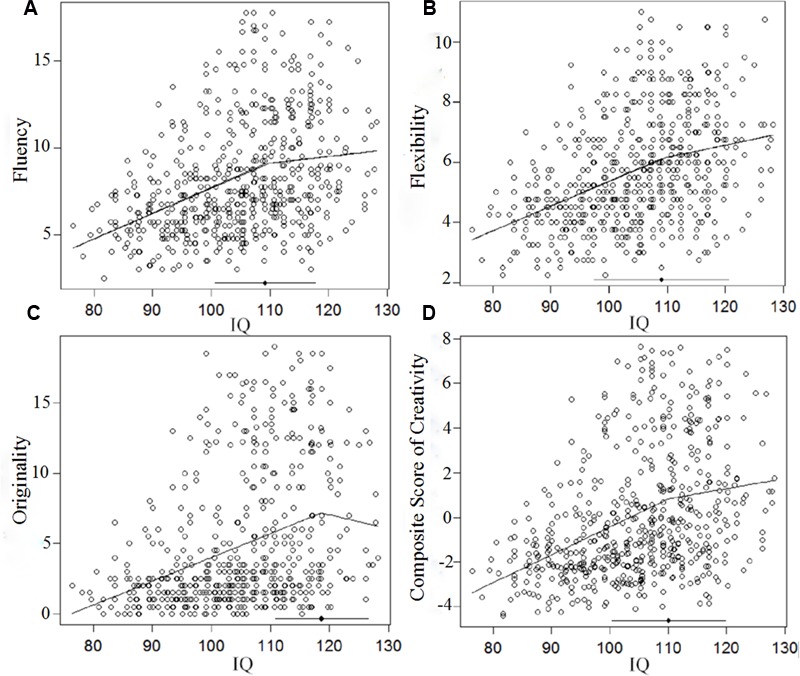
**Segmented regression analyses of the relationship between intelligence and: fluency (A)**, flexibility **(B)**, originality **(C)**, and the composite score of creativity **(D)**.

Segmented regression was conducted for fluency, flexibility, originality, and the composite score of creativity, respectively. For fluency, the breakpoint was detected at 109.20 IQ points, as shown in **Figure 1A**. The correlation between IQ and fluency below this breakpoint was *r* = 0.36(*p* < 0.01, *n* = 373). The correlation between IQ and fluency above this breakpoint was *r* = 0.05(ns, *n* = 195). The two correlations were significantly different according to Steiger’s *z*-test (*z* = 3.64, *p* < 0.01). For flexibility, the breakpoint was also detected at 109.20 IQ points, as shown in **Figure 1B**. The correlation between IQ and flexibility below this breakpoint was *r* = 0.36 (*p* < 0.01, *n* = 373). The correlation between IQ and flexibility above this breakpoint was *r* = 0.11 (ns, *n* = 195). The two correlations were significantly different according to Steiger’s *z*-test (*z* = 3.05, *p* < 0.01). For originality, the breakpoint was detected at 116.80 IQ points, as shown in **Figure 1C**. The correlation between IQ and originality below this breakpoint was *r* = 0.32 (*p* < 0.01, *n* = 486). The correlation between IQ and originality above this breakpoint was *r* = -0.09 (ns, *n* = 82). The two correlations were significantly different according to Steiger’s *z*-test (*z* = 3.40, *p* < 0.01). For the composite score of creativity, the breakpoint was detected at 110.10 IQ points, as shown in **Figure 1D**. The correlation between IQ and creativity below this breakpoint was *r* = 0.36 (*p* < 0.01, *n* = 374). The correlation between IQ and creativity above this breakpoint was *r* = -0.04 (ns, *n* = 194). The two correlations were significantly different according to Steiger’s *z*-test (*z* = 5.03, *p* < 0.01).

### Necessary Condition Analysis of DT and Intelligence

**FIGURE 2:**
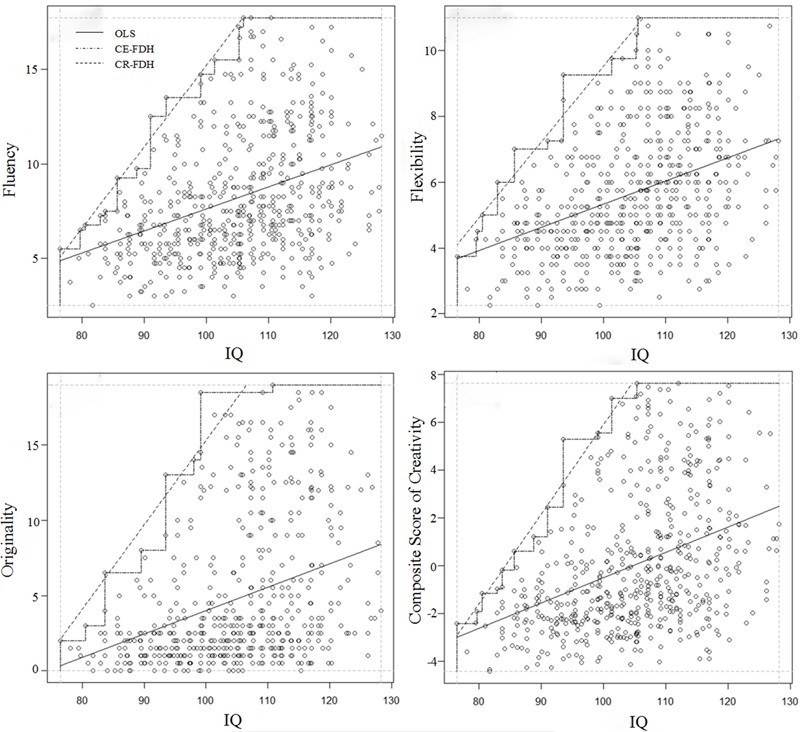
**Visualization of the NCA of the relationship between DT and intelligence**.

Necessary Condition Analysis method (Dul, 2016; Karwowski et al., 2016) was adopted, and **Figure 2** presents the results. The NCAs effect sizes (CR-FDH) are *d*_1_ = 0.235 (for fluency), *d*_2_ = 0.225 (for flexibility), *d*_3_ = 0.259 (for originality) and *d*_4_ = 0.237 (for composite score of creativity) in the interval of moderate value (0.1 ≤ *d* < 0.3 according to Dul, 2016), which statistically indicates that intelligence is a necessary-but-not-sufficient condition of creativity.

### Moderating Effect of Openness to Experience at Different IQ Levels

To examine whether the enhancing effect of openness to experience is consistent at different levels of intelligence, we divided the data into two subsamples at the breakpoint of intelligence. Then, we employed a hierarchical regression analysis to separately test the moderating effect of openness in the relationship between creativity and intelligence in the two subsamples. All predictive variables were mean centered. In Step 1, the control variables (gender and age which transformed into dummy variables) were entered. In Steps 2 and 3, the independent variable (intelligence) and the moderating variable (openness) were entered. The intelligence by the openness interaction term was entered in Step 4 to estimate the interaction effect. The results are as follows: For fluency, the interaction term is significant below the breakpoint (i.e., IQ < 109.20), with standardized regression coefficients of β = 0.14 (*t* = 2.41, *p* < 0.05) and Δ*R*^2^ = 0.01 [Δ*F*_(1,367)_ = 5.80, *p* < 0.05]. A simple effect test indicates that intelligence can predict fluency more strongly for the high openness group than for the low openness group (*ns*). However, the interaction term is not significant above the breakpoint (IQ > 109.20). Similar effects were observed for flexibility [β = 0.12, *t* = 2.06, *p* < 0.05, Δ*R*^2^ = 0.01, Δ*F*_(1,367)_ = 4.24, *p* < 0.05], originality [β = 0.13, *t* = 3.07, *p* < 0.05, Δ*R*^2^ = 0.02, Δ*F*_(1,480)_ = 9.42, *p* < 0.05] and composite score [β = 0.15, *t* = 2.73, *p* < 0.05, Δ*R*^2^ = 0.02, Δ*F*_(1,368)_ = 7.44, *p* < 0.05], which indicates that the moderating effect of openness is significant below the breakpoints but not above the breakpoints.

## Discussion

### Threshold Effect

The threshold hypothesis suggests that a threshold exists between creativity and intelligence. According to Guilford and Christensen (1973), positive relations between intelligence and creative ability exist when the IQ is below the threshold, whereas no correlation exists when the IQ is above the threshold. By means of testing and questionnaire methods, segmented regression and NCA, this study tested this hypothesis and identified a breakpoint in the correlation data between IQ and creativity. Below this breakpoint, the correlation between intelligence and creativity is stronger; however, it weakens when intelligence is above the breakpoint. Our findings are similar to those of previous studies, including the studies by Barron (1963, 1969), Jauk et al. (2013), Karwowski and Gralewski (2013), and Karwowski et al. (2016). To a certain extent, these results conform to the classical threshold hypothesis and demonstrate that high creativity in terms of DT requires high intelligence or above-average intelligence. However, this condition is only a necessary and insufficient condition for high creativity, which confirms that intelligence and creativity are two overlapping variables, as proposed by Renzulli (1978) and summarized by Sternberg and O’Hara (1999). Considering that Gf is the ability to use mental operations to reason and solve problems and has a closer relationship with nature than nurture (Sawyer, 2012), we can understand this limited correlation between intelligence and DT. High creative ability requires acquired experience in addition to gifted talent.

However, the turning point in our study is different. Guilford and Christensen (1973) assumed that the break IQ level is approximately 120. Karwowski and Gralewski (2013) observed a threshold of 115 IQ points. Jauk et al. (2013) observed a threshold of 119.60 IQ points for originality and a threshold of 86.09 for fluency. In this study, the breakpoint ranged from 109.20–116.80, with an IQ of 109.20 for DT fluency and flexibility, an IQ of 116.80 for originality, and an IQ of 110.10 for the composite score of creativity. Some possible explanations for the difference between the results of previous studies and the results of this study are presented: First, this study investigates the threshold hypothesis in elementary school students, whereas previous studies investigated the threshold hypothesis in preschoolers (Getzels and Jackson, 1962; Barron, 1963, 1969; Fuchs-Beauchamp et al., 1993), secondary school children (Karwowski and Gralewski, 2013), adolescents and adults (Cho et al., 2010; Jauk et al., 2013). Because intelligence and creativity are associated with age, when we explore the relationship between intelligence and creativity with subjects of different ages, we may obtain different results. These findings also indicated the need to conduct a study with elementary children. Second, differences between our study and previous studies concerning cultural background were identified. Compared with children in Western cultures, Chinese children have a disadvantage in terms of creativity (e.g., Ye et al., 1988; Hu et al., 2004); however, this situation does not exist in terms of intelligence (e.g., Lynn et al., 1988, 1991). This finding indicates that intelligence may explain less variance in creativity for Chinese children for high IQ levels. As Csikszentmihalyi (1999) noted, creativity is a culturally bound phenomenon and is not a mental process. When describing a creative person, Western implicit concepts focus on a person’s motivational qualities, personality characteristics and cognitive traits. These traits include high IQ level, logical and skillful problem solving, clear thinking and cleverness, as emphasized by parents and teachers (Runco et al., 1993). Unlike Western concepts, Chinese implicit conceptions of creativity emphasize characteristics such as moral goodness, societal contributions and connections between the new and the old (Rudowicz and Yue, 2000; Niu and Sternberg, 2002). Chinese culture places less emphasis on intelligence than Western culture with regard to creativity, and high intelligence accounts less for the variance in creativity in China. This finding may explain why the IQ thresholds in this study are lower than the IQ thresholds in previous studies. As we expected, one finding of this study is that the threshold of intelligence for Chinese children is lower than the threshold of intelligence for Western children, which is typically considered to be 120 points according to Guilford and Christensen (1973) or 119.60 points according to Jauk et al. (2013) and 115 points according to Jauk et al. (2013). These different thresholds of intelligence suggest that people should pay more attention to other factors in addition to intelligence when enhancing creativity in China.

### Moderating Effect of Openness to Experience

Many previous studies have indicated openness as the most important factor of personality in creativity (Feist, 1998; Furnham, 1999; Chamorro-Premuzic and Furnham, 2004). According to Batey et al. (2009), openness to experience (and extraversion) enables people to be more curious, experiential and interested in “quirkiness,” which may increase their ability to produce new ideas. King et al. (1996) examined the relations among personality, creative ability and creative accomplishments. They determined that openness served a “catalyst” role in the association between verbal creativity (as measured by DT) and creative accomplishments. When openness to experience was at a high level, people with high verbal creative ability reported relatively more creative accomplishments. This moderating effect of openness was also discussed in recent studies (e.g., Ivcevic and Brackett, 2015; Shi et al., 2016). In their exploration of the relationship between the threshold hypothesis and the Big-Five Structure Inventory, Jauk et al. (2013) discovered that creative potential is significantly predicted by IQ and conscientiousness below the IQ threshold. Above the IQ threshold, openness to experience is currently the strongest predictor of creative potential; intelligence and conscientiousness are only significant predictors by trend. These findings indicate that openness to experience is a personality factor that may moderate the relationship between intelligence and creativity. Is this moderation effect consistent at different levels of intelligence? To answer this question, we employed a hierarchical regression analysis to test the moderating effect with two subsamples (below and above the breakpoint of intelligence). The results indicated that the moderating effect of openness only exists below the threshold level of intelligence; openness only served the “catalyst” role in the association between intelligence and creative thinking when the IQ was average or low. Based on this result, the role of personality factors appears to be conditional and depends on the level of cognitive ability.

### Limitations and Future Directions

Several limitations of this study should be acknowledged. As Runco and Albert (1986) suggested, thresholds have contradictory and inconclusive results due to the different measures of intelligence and creativity and the highly selective samples that were employed in previous research. Only fluid intelligence (Gf; measured by RPM) and DT (measured by TTCT) were employed in the current study, which may impact the results of the threshold hypothesis. Future studies should employ multiple measures to assess participants’ intelligence and creativity. Moreover, Welter et al. (2016) suggested that the association between intelligence and creativity was not straightforward and was dependent on a combination of factors, including grade level and gender. Another limitation of our study is the lack of gender balance in the sample; future research can explore the threshold hypothesis with more balanced samples.

## Conclusion

This study investigated the relationship between creativity and intelligence with Chinese children. The current results support the threshold theory demonstrating that intelligence is a necessary-but-not-sufficient condition of creativity. Specifically, the breakpoints around 110 IQ level was detected. In addition, openness to experience had a moderating effect on the correlation between the indicators of creativity and intelligence under the breakpoint. Above this point, however, the effect was not significant. It remains necessary to explore the personality factors accounting for individual differences in the relationship between DT and intelligence.

## Author Contributions

BS designed the study and performed the investigation; LW and JY analyzed the data, BS, MZ, and LX wrote the manuscript.

## Conflict of Interest Statement

The authors declare that the research was conducted in the absence of any commercial or financial relationships that could be construed as a potential conflict of interest.
